# The challenge of adopting a collaborative information system for independent healthcare workers in France: a comprehensive study

**DOI:** 10.1038/s41598-024-62164-2

**Published:** 2024-05-19

**Authors:** Laurent Gaucher, Céline Puill, Sophie Baumann, Sophie Hommey, Sandrine Touzet, René-Charles Rudigoz, Marion Cortet, Cyril Huissoud, Pascal Gaucherand, Corinne Dupont, Frédéric Mougeot

**Affiliations:** 1 Pôle de santé publique, Hospices Civils de Lyon, 69500 Bron, France; 2 Research on Healthcare Performance (RESHAPE), INSERM U1290, Université Claude Bernard Lyon 1, 69008 Lyon, France; 3 GENeva MIdwifery Research unit, Geneva School of Health Sciences, HES-SO University of Applied Sciences and Arts Western Switzerland, Geneva, Switzerland; 4 Independent Midwife, 94120 Val de Marne, France; 5 Midwifery Department, EA 7285, Versailles Saint Quentin University, 78180 Montigny-le-Bretonneux, France; 6 Department of Obstetrics and Gynecology, Poissy-Saint Germain Hospital, 78300 Poissy, France; 7 Hospices Civils de Lyon, Hôpital de la Croix-Rousse, 69004 Lyon, France; 8 Hospices Civils de Lyon, Hôpital Femme Mère Enfant, 69004 Lyon, France; 9 Réseau Périnatal Aurore, 69004 Lyon, France; 10 Centre Max Weber, UMR 5283, Institut des Sciences de l’Homme, 69363 Lyon, France

**Keywords:** Health services, Epidemiology

## Abstract

This study aimed to explore the perception of an underutilised collaborative information system through qualitative research, utilizing semi-structured, in-depth interviews with independent midwives and physician. PROSPERO, is a collaborative information system designed to bridge the communication gap between community-based healthcare workers and hospital-based care teams for parturients in Lyon, France. Through 27 semi-structured in-depth interviews with midwives, obstetricians, and general practitioners, we identified key themes related to the system’s adoption: implementation challenges, utilisation barriers, interprofessional dynamics, and hidden variables affecting system use. Participants recognised the potential of PROSPERO to improve information sharing and care coordination but expressed concerns about the system’s integration into existing workflows, time constraints, and the need for adequate training and technical support. Interprofessional dynamics revealed differing perspectives between hospital and independent practitioners, emphasising the importance of trust-building and professional recognition. Hidden variables, such as hierarchical influences and confidentiality concerns, further complicated the system’s adoption. Despite the consensus on the benefits of a collaborative information system, its implementation was hindered by mistrust between healthcare workers (i.e. between independent practitioners and hospital staff). Our findings suggest that fostering trust and addressing the identified barriers are crucial steps towards successful system implementation. The study contributes to understanding the complex interplay of factors influencing the adoption of collaborative healthcare technologies and highlights the need for strategies that support effective interprofessional collaboration and communication.

*ClinicalTrials ID* NCT02593292.

## Introduction

Antenatal care models worldwide are shaped by diverse cultural, systemic, and professional paradigms, leading to varied dynamics between midwives and physicians. Building on Buchet and Strauss’s^1^ concept of evolving professional segments through internal movements and conflicts, current perinatal care often embodies both collaboration and contention. This dynamic is exemplified in the Swiss context by Cavalli and Gouilhers (2014) and in Turkey by Topçu (2019), who reveal the paradoxical trend of ‘vaginarean’ births^2,3^. These are vaginal deliveries conducted with the extensive use of medical technology, similar to caesarean sections, yet paradoxically viewed as risky^2^. Cavalli and Gouilhers^3^ also highlight tensions between midwives and doctors in Switzerland, pointing to conflicts over competency recognition. Despite scientific advocacy for midwife-led models by Sandall et al.^4^, these complex interprofessional interactions require a nuanced understanding to enhance prenatal care. France’s unique healthcare system provides a pertinent backdrop to explore these global insights.

**Figure 1:**
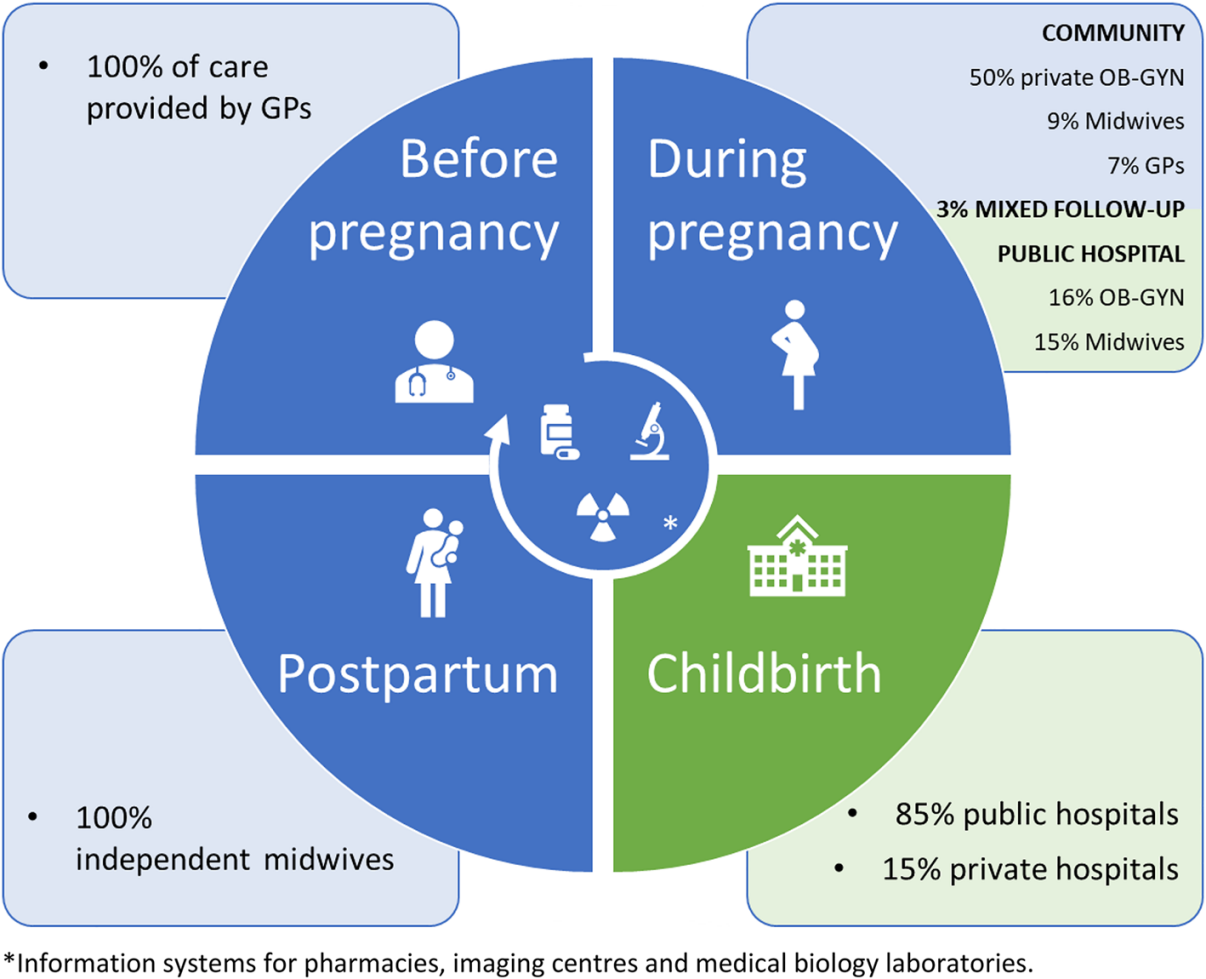
Perinatal health care with diverse healthcare workers and their distinct medical information systems in France (2016, before intervention).

In 2021, France reported a fertility rate of 1.84 live births per woman, marking the highest among European Union member states and highlighting its unique standing in the field of childbirth and perinatal care within the European Union. This statistic positions France at the forefront of European nations in terms of birth rates, indicative of its strong healthcare infrastructure and the socio-cultural factors that influence family planning decisions. Despite witnessing approximately 723,000 births in 2022, France is observing a gradual decline in birth numbers compared to previous years^5^. The French healthcare system is characterised by a professional segmentation not only among healthcare workers but also between private and public sectors, as well as between community practices and hospital or clinic-based care. As Fig. 1 illustrates, while 50% of antenatal care is administered by private obstetrician-gynaecologists, a majority of childbirths take place within the public sector, predominantly in public hospitals^6,7^. This duality within the system, reflecting both Beveridge and Bismarckian models, underscores the urgency for a unified collaborative information system to bridge the divide, improving coordination and integration across different care settings^8^.

To reduce the communication gap between community care and hospital services in the French healthcare system, we initiated and implemented the Program of Research for the Optimisation of a Supervised Parturient Pathway for Expectant Women according to their Risk in Obstetrics (PROSPERO). The endeavour to foster a collaborative information-sharing framework among independent healthcare workers presents a multifaceted challenge, necessitating robust interprofessional cooperation and dialogue between healthcare workers and information technology specialists^9,10^. To address these challenges, the PROSPERO programme, collaboratively designed and developed with community healthcare workers, hospital staff, and service users, sought not only to optimise the care pathway for expectant women but also to enhance medical information sharing among healthcare workers. PROSPERO is a web-based platform to streamline the medical data sharing among community providers, hospital personnel, and women. The platform proposed a tailored clinical route, predicated on early pregnancy medico-psycho-social risk assessment in hospitals, aligning with hospital caregivers’ view of pregnancy as inherently risky^11^.

We crafted PROSPERO with four primary structured form categories: Staturo-Ponderal Evolution, Vitals, Functional Signs, and Obstetric and Pelvic Exam, complemented by a free text field for detailed notes and consultation actions. Pre-tests revealed that completing the form takes 3 to 10 min. The platform’s dual SMS authentication, while extending login time marginally, significantly enhances data security. Community healthcare workers initially faced challenges adjusting to the new workflow, particularly when integrating with existing software. However, the secure data hosting on the Hospital Civils of Lyon’s servers ensures strict adherence to French and European data protection regulations.

The programme’s pilot phase, focusing on reliability testing without comprehensive evaluations from healthcare workers or women, places it in the early stages of the Clinical Adoption Meta-Model (CAMM), specifically in the “Preparation” and “Exploration” phases^12^. During these stages, the emphasis is on understanding the technology’s potential, ensuring its reliability, and identifying the initial reactions from potential users. The core components and objectives of PROSPERO are presented in Table 1 according to HITO (Health Information Technology Ontology) recommendations^13^. Despite collaboration with healthcare workers across sectors, the uptake by community providers was minimal, prompting us to involve a sociologist for an in-depth qualitative analysis to uncover the reasons for the limited use of the PROSPERO programme.

**Table 1:** Description of PROSPERO aligned with HITO classes.

Aspect	Description of PROSPERO
Software product	Programme of research for the optimisation of a supervised parturient pathway for expectant women according to their risk in obstetrics (PROSPERO)
Feature	1. Risk assessment tools for healthcare workers and pregnant women 2. Secure information-sharing capabilities including consultation reports, ultrasound images, and lab results 3. Tools for shared clinical pathway management
Application system type	Web-based health information system
Organisational unit	Maternity care departments within community and hospital settings
User group	Midwives, general practitioners, specialists, and pregnant women—both community and hospital-based

The primary aim of this study is therefore to understand this failure by exploring the perception of this collaborative information system through a qualitative study that collected data using semi-structured in-depth interviews of independent midwives and physicians.

## Methods

All interviews were conducted by a male sociologist (FM) with a PhD, specialised in the sociology of work and health, collaborating with our research team. He had experience in the health field (direct observations in psychiatric wards, conducting focus groups with cystic fibrosis patients and healthcare workers) and has conducted many interviews with various healthcare workers, but had no experience in the field of midwifery or obstetrics. The sociological study was supplemented by a double reading of some of the interviews by a woman midwife trained as a health anthropologist (CP). As an independent midwife, she has expertise in the field of midwifery and obstetrics that complements the interviewer’s expertise in the sociology of work and health. The coordinating investigator (LG), a male hospital midwife, with a PhD in public health and training in qualitative research in health care, participated with them in discussions on the interpretation of interviews, based on anonymised interview extracts.

The interviewer (FM) and the second analyst (CP) had no relationship with the participants before this study. Interviewees were informed that their interviewer was a sociological researcher employed to report their experience using the collaborative information system. Grounded theory was used to generate theory through data from independent and hospital healthcare workers^14^.

The interviews were conducted 9 to 12 months after the initial deployment of the PROSPERO software and approximately three months prior to the recognition of its underperformance issues. We recruited healthcare workers from the information exchange platform (MonSisra) for information sharing between healthcare workers. Participants who were independent practitioners involved in antenatal care outside hospitals (general practitioners, medical gynaecologists, gynaecologist-obstetricians, and midwives) and provided care for pregnant women included in the PROSPERO trial met the study inclusion criteria. Our non-probabilistic, purposive sampling approach called for the selection of extreme or deviant cases and maximum variation sampling^15^. At the same time, the selection process aimed to achieve representativeness regarding the age and diversity of status of the professionals surveyed as well as to include those with much lower or higher participation than average in this exchange platform. Appointments were made in advance. The coordinating midwife (LG) and the investigating physician (RCR) facilitated the interviews by introducing the sociologist (FM) to the healthcare workers by mail, e-mail, or in person.

We aimed to conduct up to 30 interviews, with the process guided by grounded theory principles^16^. Interviews, mainly conducted during healthcare workers’ working hours, were designed to be exploratory and semi-structured, based on the six dimensions outlined in Table 2. The process was adaptable, with the potential to conclude early if data saturation was achieved, as determined by the sociologist^17^. The data collection was stopped when the theoretical framework no longer evolved under the influence of new data, reflecting grounded theory’s non-sequential approach to data analysis where hypothesis formation, data collection, and analysis occur concurrently, rather than in a step-by-step manner. Each interview was unique to the participant, recorded with consent, and the content was carefully transcribed and analysed for accuracy and insight.

**Table 2:** Analytical framework to understand healthcare workers’ perspectives in the PROSPERO study.

Dimension	Description	Key Questions
Participant’s career path	Exploring the healthcare workers’ journey into their current role, emphasising the transition to independent practice	Could you tell me about your career history?
Relationships: parturient/community/hospital	Assessing the dynamics between the healthcare worker, their patients, and the hospital, focusing on pregnancy care	In the context of pregnancy care, what are your current links with the hospital?
Attitude towards ICTs	Understanding the role and significance of ICT in their professional activities	How important is the use of ICT in your work?
Relationship with the hospital	Gauging the nature of the healthcare workers’ relationship and interactions with hospital staff	How would you describe your relationship with the hospital?
Collaborative information system	Investigating the methods and practices for information exchange about parturient women among healthcare workers	How do you exchange information about a woman with your colleagues?
Outstanding issues	Providing an open-ended opportunity for participants to discuss additional topics or concerns not covered in the interview	Is there anything I haven’t mentioned that you’d like to discuss?

*ICT* Information and Communication Technology.

Data analysis took place as the data was collected. Further comparative analyses were conducted after each round of interviews. All transcripts were reviewed manually (no software was used). The decision to forgo verbatim analysis software stemmed from the lead sociologist’s methodological approach, prioritising in-depth, interpretive analysis of interview content. This choice reflects a focus on qualitative richness and contextual in-depth understanding of participants’ perspectives, aligning with the study’s exploratory aims. More than the quantification of a theme, which has no statistical value, it is the meaning given by the actors to their experience and actions that has been considered, independently of the number of occurrences. This approach was essential in order to gain an analytical and not just a descriptive perspective. The transcript was first reviewed by the interviewer who used open-coding to identify emerging concepts related to perceptions of the collaborative information system. This text-based coding assigned a theme to each speech segment. After the initial open-coding of all interviews, i.e., the assignment of new themes to each text segment, the codes and related transcript excerpts were examined and grouped into similar themes, according to a coding tree. Thus similar minor themes were grouped into major themes, as conceptualised in the scientific literature^18^. The themes were not identified in advance, but were derived from the data.

A second member of the research team (CP) then used her own open-coding to independently analyse six of the transcripts, selected by the first coder for their relevance to and richness regarding the subject matter of the research. The concepts and associated definitions of these separate codes were then merged into a jointly developed coding scheme^19^, and areas of disagreement resolved through in-depth discussions.

Obstetricians or midwives had provided women with information about the trial during the first prenatal visits at hospital. Individual consent was obtained from all healthcare workers participating of this study. In accordance with current French legislation, this study was reported to the French commission for information technology and civil liberties (CNIL; registered number: HCL 18-165). Further, it was approved by the French ethics research committee Ouest VI under the authority of the French Directorate General for Health (approval number: CPP 1084 RNI). This study was also registered at the ClinicalTrials website under ID NCT02593292. This study was reported in accordance with the Consolidated criteria for reporting qualitative research (COREQ) guidelines.

### Ethical approval

Obstetricians or midwives provided women with information about the trial during the first prenatal visits at hospital. Women confirmed participation and provided written informed consent. Individual consent was obtained from all participants of this study. In accordance with current French legislation, this study was reported to the French commission for information technology and civil liberties (CNIL; registered number: HCL 18-165). Further, it was approved by the French ethics research committee Ouest VI under the authority of the French Directorate General for Health (approval number: CPP 1084 RNI). This study was also registered at the ClinicalTrials website under ID NCT02593292.

## Results

### Sample characteristics

Our initial sample included 30 participants but 3 obstetricians were ineligible because they did not use the collaborative information system. We therefore analysed the interviews of 27 participants: 22 midwives (including 2 males), 3 obstetricians (2 females and 1 male), and 2 general practitioners (1 male and 1 female). Saturation was reached at this point according to FM. The duration of the interviews ranged from 35 to 75 min, with a majority lasting around 50 min. The mean age of the study population was 41.5 years.

We identified three major themes and eight subthemes related to the use/adoption of the collaborative information system through thematic analysis of the interviews (Table 3).

**Table 3:** Major themes and subthemes identified through thematic analysis.

Major themes	Subthemes	Description
1. Implementation challenges	Necessity of a collaborative information system	Exploring the perceived need for the collaborative information system among healthcare workers
	Work habit changes	The difficulties healthcare workers face in altering established work routines to accommodate the new system
2. System utilisation barriers	Time constraints	Concerns about the time required to effectively use the system within the confines of existing consultation structures
	Training and technical issues	The lack of adequate training for healthcare workers and technical limitations of the system hindering its use
3. Interprofessional dynamics	Hospital vs. Independent practice	The differing perspectives and practices between hospital-employed and independent healthcare workers
	Cure vs. Care	The dichotomy between the clinical, intervention-focused approach (cure) and the holistic, patient-centred approach (care) within the collaborative information system context
	Professional recognition	The quest for acknowledgment and respect among different healthcare professions and settings
4. Hidden influences	Hierarchical influences	The influence of hierarchical structures on system adoption, revealing how power dynamics shape professional autonomy and interprofessional relationships
	Funding and resources	The role of financial support and resource allocation in the system’s implementation and use
	Confidentiality concerns	The importance of maintaining patient privacy and confidentiality in collaborative information systems

### Theme 1: implementation challenges

#### Necessity of collaborative information system

The introduction of a collaborative information system is perceived as a significant advancement by healthcare workers. A general practitioner reflects on the potential benefits: *“If I had software that would be straightforward and where, at the end, it’s as if my patient had been seen by another midwife or another physician at the hospital, that would be amazing”* (General practitioner, in his 40 s, independent).

#### Work habit changes

Adapting to the new system requires healthcare workers to modify their established work routines, a process which is not without challenges. One midwife candidly shares: *“But when there are particular problems, it’s true you don’t have to note it; if the baby’s in intensive or special care, and why, that’s written on the health booklet, but then, you’d have to read it, you’d have to ask questions, and that’s work”* (Midwife, in his 40 s, independent). This highlights the effort required to fully engage with the new system and utilise its potential effectively. Despite the challenges, healthcare workers recognize the potential of the collaborative information system to enhance communication and coordination in patient care. Another midwife notes: *“Most of the time, it’s more efficient to use the phone. Afterwards, there is everyone’s email addresses. The way it’s structured, it’s quite easy when you know their names and their first names, to find their email addresses.”* (Midwife, in her 50 s, independent), suggesting that while the system delivers upon its promise, existing methods of communication still play a crucial role.

### Theme 2: system utilisation barriers

#### Time constraints

Healthcare workers expressed concerns about the time-consuming nature of integrating the new system into their workflow. One midwife highlighted the duplicative nature of tasks, stating*, “The hospital midwife who sees my patient is already taking her history, and all that history, if she sent it to me, I wouldn’t have to do it again. It would save me an incredible amount of time”* (Midwife, in her 50 s, independent). Another midwife elaborated on the process, *“We midwives do half-hour consultations. … If everything goes well and it’s quick, in 20 min, it can be done. And afterwards, I will take the time … I have to transform [my consultation report] into PDF, and send it to My Documents, then go to … my [professional software] account, then the patient’s account, [back to] My Documents, to transmit the PDF. It takes me an incredible amount of time”* (Midwife, in her 50 s, independent).

#### Training and technical issues

The lack of training and technical support was another significant barrier to the system’s effective use. One midwife suggested, *“Maybe there should be training for professionals as well… and show us that it works, that it functions, that it’s easy, that it doesn’t take time”* (Midwife, in her 50 s, mixed hospital-independent practice). Technical limitations also posed challenges, as another midwife pointed out, *“We’re limited in the size of the attachments. So, depending on how we scanned them or not, not all attachments fit”* (Midwife, in her 50 s, independent).

### Theme 3: interprofessional dynamics

#### Hospital vs. Independent practice

Healthcare workers from independent practices often perceive a divide in professional respect and acknowledgment compared to their hospital-employed counterparts. One independent midwife expressed, *“There are a lot of [hospital healthcare workers] who think that the real midwives are in the hospital, and that as an independent, it’s… it’s sub… [we’re] sub-midwives.”* (Midwife, in her 50 s, independent). This sentiment was echoed by an obstetrician who felt marginalized by hospital processes, stating, *“The hospital doesn’t need us, that’s the way we feel. But we need to know what’s going on in the hospital.”* (Obstetrician, in her 60 s, mixed hospital-independent practice). The lack of communication and collaboration between these two sectors was a recurring concern, with one midwife regretting the absence of information sharing by the hospital*, “Given modern means of communication [and that] she was first seen at the hospital, they could send me a letter saying, ‘you’re going to have this patient to follow up, here’s the history, here’s what we did.’ No, they don’t do that.”* (Midwife, in her 50 s, independent).

#### Cure vs. care

A tension between the technical, medical aspects of healthcare (cure) and the supportive, preventative side (care) was also highlighted. An obstetrician commented on the collaborative information system’s limitation in transmitting medical instructions, reflecting a broader issue in the interprofessional dynamics, *“Finally, it is important to note that the software was built to allow the exchange of information about women’s care and situation, but not to transmit medical instructions. Their profession [speaking about midwifery] is one of support, it’s not a profession of medical decision-making.”* (Obstetrician, female, in her 60 s, mixed practice).

#### Professional recognition

The quest for professional recognition was a significant concern among independent healthcare workers. The feeling of being undervalued or not fully recognized by hospital counterparts was a common theme. This segment also highlights the dissatisfaction of independent healthcare workers with tasks they deem menial, such as floor cleaning or filling out collaborative information system, further contributing to their sense of underappreciation. A midwife shared how her frustration of her work at the hospital was what led her to become independent, *“I didn’t study midwifery to type on a computer or mop floors. I didn’t like it any more. So, I decided to go into independent practice, where I’m devoted solely to my patients.”* (Midwife, in her 50 s, independent). The desire for a more collaborative and respectful relationship between hospital and independent healthcare workers was evident, with one midwife emphasising the importance of direct communication with women, *“We midwives usually have half-hour consultations. I am there for the woman, to talk with her, to respond to her.”* (Midwife, in her 50 s, independent).

### Theme 4: hidden influences

#### Hierarchical influences

The hierarchical structure within healthcare settings can greatly affect the flow of information and collaboration. For example, *“I have always found it difficult, the relationship with midwife-managers, with the whole pyramidal and hierarchical system of the hospital. I wasn’t comfortable with that. And I found that as an independent practitioner, you’re really in control, you work as you want, you do what you want…”* (Midwife, in her 30 s, independent). This quote highlights how hierarchical pressures can drive healthcare workers towards independent practice, seeking autonomy over their work. A tool, even collaborative, imposed by the hospital could give them the feeling of returning to hierarchical dependency.

#### Funding and resources

The availability of funding and resources is a critical yet often overlooked aspect that can determine the success or failure of implementing new systems. The need for adequate support is encapsulated in the frustration of a midwife: *“If I had a secretary, I could ask her to do it, but I don’t have a secretary, I’m on my own. So once, twice, three times and after a while I stopped because it was too complicated.”* (Midwife, in her 30 s, independent). This statement underlines the challenges healthcare workers face when expected to manage additional administrative tasks without the necessary support infrastructure or financial resources.

#### Confidentiality concerns

Confidentiality remains a paramount concern in the healthcare sector, especially when transitioning to a collaborative information system. The delicate balance between sharing necessary information and protecting patient privacy is articulated by a midwife*: “I don’t enjoy writing on her file that the patient was raped when she was 15 years old: ‘difficulties, visits her shrink*+++*’. I can’t do it. I’m not gonna write that on her chart. If she sees it, she’s going to think the midwife said that something was wrong.”* (Midwife, in her 20 s, independent). This quote illustrates the ethical dilemmas healthcare workers face when documenting sensitive information in a collaborative information system.

## Discussion

Drawing from interviews with 27 healthcare workers, our findings emphasise the importance of system reliability and comprehensive training, aligning with DeLone and McLean’s dimensions of system and information quality. The imperative to tailor technology to the varied needs of healthcare roles, as depicted in our “Hospital vs. Independent practice” and “Cure vs. Care” themes, mirrors the “Individual-Technology Fit” concept of the FITT framework. Furthermore, information sharing was perceived as a subordinate (i.e. menial) task, highlighting the entrenched professional divisions between independent and public hospital care in France, ultimately impacting patient care.

### Interpretation

Our investigation aligns with the “System Quality” and “Information Quality” dimensions of DeLone and McLean’s model, highlighting the necessity for effective and reliable collaborative information systems in obstetrics^20^. Challenges were revealed in our study, such as “technical limitations” and “insufficient training”; that accentuate the need for proficient systems and precise, relevant data to facilitate successful adoption of healthcare Information Technology (IT). In accordance with the “Individual-Technology Fit” of the FITT framework, our findings, particularly “Hospital vs. Independent practice” and “Cure vs. Care”, illustrate the critical importance of tailoring technology to the diverse requirements and practices of healthcare workers^21^. Echoing prior studies, research from the United States (US) revealed that vital clinical care details, such as social health determinants, frequently escape documentation in electronic medical records (EMRs) due to their designs not tailored to accommodate such information^22^. Also in the US, healthcare workers identified the complexity and time-consuming aspect of consulting EMRs as major obstacles in a 2015 study^23^. Community clinics in New York faced challenges related to time, staffing, and funding, which subsequently restricted administrative support for EMR usage and access to training^24^.

Our study also introduces innovative themes such as “Interprofessional dynamics” and “Hidden influences” like “Professional recognition” and “Hierarchical influences” extend beyond traditional IT adoption frameworks, suggesting the complexity of sociotechnical interactions in healthcare settings, which may not be fully captured by existing models like those of DeLone and McLean or the FITT framework^20,21^.

The sociological insights of Florent Champy resonate profoundly in our study, particularly his concept of “prudential practice” within health professions. Champy articulates that these practices necessitate practical judgment (phronesis) in the face of complex and uncertain situations, tailored to individual users^25^. This perspective is mirrored in our findings, especially within themes such as “Cure vs. Care” and “Confidentiality concerns”. Our data suggest that our collaborative information system, though intended to enhance care, may inadvertently hinder the exercise of phronesis. Factors such as bureaucratisation, division of labour, an emphasis on performance and objectivity, and the erosion of professional autonomy appear to complicate the practical judgment essential in healthcare.

Moreover, the lukewarm reception of our collaborative information systems among healthcare workers may be partially attributed to the traditional notion of “professionalism”. This concept posits that professional groups strive to develop and maintain exclusivity within their domain to safeguard their interests, such as salary, status, power, and the monopolistic protection of their professional jurisdiction^26^. The rise of independent midwifery in France, where the involvement of midwives in pregnancy follow-ups increased from 8.5% in 2016 to 22.9% in 2021, primarily at the expense of private practice gynaecologists whose share decreased from 49.7% to 39.4%, highlights increasing interprofessional tensions^7^. This shift raises significant questions related to our sub-themes of “Professional recognition”, and “Funding and resources”. These barriers to collaborative practice are not unique to obstetrics; a study involving 229 healthcare workers across various specialties in Lahore identified role ambiguity, divergent individual team member goals, and differences in authority levels, expertise, and income as the main obstacles to effective interprofessional collaboration^27^.

Finally, these tensions between independent and hospital-based healthcare workers over information transmission reflect different values attributed to healthcare work. They also reveal a vision of this work of information provision as menial^28,29^. As Hughes argued, work is composed of a more or less noble set of tasks to be accomplished. The core of this bundle of tasks is the prestigiousness or honourability of the work, while the subordinate or peripheral—the menial—tasks can be delegated to others^30^. In our study, some midwives considered listening to women as the noble part of their work, while doctors put more emphasis on decision-making. But both professions and both segments of each (independent ambulatory and hospital sectors) appeared to treat the use of information transmission collaborative information system as a menial task. This most likely explains why its implementation failed.

### Strength and limitations

The main strength of this study is the ethnographic approach’s ability to uncover deep insights into the users’ perceptions and experiences with PROSPERO, providing a rich understanding of the complex interplay of social, organisational, and technological factors affecting the platform’s adoption. A sociologist previously uninvolved in this topic and without prior knowledge—and therefore preconceptions—about care providers specialised in the perinatal period conducted and analysed the interviews. He considered that data saturation was reached, and the final sample size was aligned with sample sizes recommended for qualitative methods^16,32^. Then, an additional analysis was entrusted to an independent midwife not part of the system under study. The diversity of the researchers (including a male sociologist, a female independent midwife, and both male and female obstetricians and hospital midwives, with various levels of education) enabled triangulation and reduced the risk of potential for bias.

While the study’s insights are valuable, it’s important to acknowledge limitations. The reliance on the lead sociologist’s judgment for data saturation and the absence of verbatim analysis software could introduce subjectivity, potentially impacting the analysis’ comprehensiveness. Additionally, the skewed distribution of participants, with a predominance of midwives over doctors, may limit the generalisability of findings across the broader healthcare worker spectrum. It provides only an overview during an ephemeral phase of a failed implementation. Moreover, although independent obstetricians provide antenatal and postnatal care for many pregnancies, they accounted for only a small part of our sample because only half of those who agreed to meet us were using the collaborative information system.

### Implications

The findings from our study underscore a critical need for a re-evaluation of the design and implementation strategies of collaborative information systems in obstetric care. To address the technical and training challenges identified, there is an imperative for developers and healthcare administrators to foster systems that are not only technically reliable but also intuitive and accommodating to the diverse needs of healthcare workers. This may include enhancing system features such as single sign-on capabilities, streamlined data entry through voice-to-text functionalities, and advanced data analytics to provide actionable insights for healthcare providers. This entails a shift towards more user-centric designs that prioritise ease of use and relevance of information, potentially through iterative co-design processes with healthcare workers. The sociotechnical complexity uncovered in our themes, such as “Interprofessional dynamics” and “Hidden influences”, requires embedding sociological insights into IT adoption strategies. For instance, including tools for automatic data synchronization between community and hospital care software to reduce redundancy and ease the workflow for healthcare providers. This could imply the development of policies and practices that recognise and mitigate the effects of professional hierarchies and segmentation, fostering a culture of mutual respect and understanding across different healthcare roles.

Moreover, the concept of “professionalism” and the protective stance of professional groups towards their domain should be carefully navigated. Engaging healthcare workers in meaningful dialogues about the benefits of collaborative systems, beyond mere information-sharing, could help in aligning the system functionalities with their professional values and practices. Emphasizing the role of the system in supporting clinical decision-making and patient management might also diminish resistance to technology adoption. This approach could mitigate the perception of information transmission as a “menial task” and reframe it as an integral part of quality patient care.

Furthermore, the incorporation of “prudential practice” into the system design, by allowing for the documentation and recognition of nuanced patient care practices, could enhance the system’s utility and acceptance. Enhanced customizability and the integration of decision support tools could further validate the clinician's expertise in the caregiving process. Finally, addressing the barriers to collaborative practice identified, such as role ambiguity and divergent goals, through targeted interprofessional education and collaborative workshops, could improve the adoption and effective use of collaborative information systems in obstetric care. The establishment of a dedicated support and liaison team might also provide ongoing assistance and promote a seamless integration of the system into daily clinical routines.

Importantly, integrating artificial intelligence (AI) and natural language processing (NLP) for voice analysis could play a transformative role, particularly in the detection of depression—a leading cause of maternal mortality in high-income countries. By analysing voice intonation patterns, AI can assist in identifying early signs of depression, thereby facilitating timely intervention and support^33^.

## Conclusion

Our study provides valuable insights into the challenges and dynamics of implementing a collaborative information system in obstetric care, highlighting the need for systems that not only cater to the technical aspects of healthcare delivery but also respect and integrate the diverse professional practices and values within the field. The findings underscore the importance of designing user-centric systems that facilitate seamless communication and collaboration among healthcare workers, thereby enhancing the quality of antenatal care.

## Data Availability

The data that support the findings of this study are available from Frédéric Mougeot (frederic.mougeot@gmail.com) upon reasonable request.
